# Cancer-Derived Extracellular Vesicles as Biomarkers for Cutaneous Squamous Cell Carcinoma: A Systematic Review

**DOI:** 10.3390/cancers14205098

**Published:** 2022-10-18

**Authors:** Irene Tai-Lin Lee, Chin-Hsuan Shen, Feng-Chiao Tsai, Chun-Bing Chen, Kevin Sheng-Kai Ma

**Affiliations:** 1 Center for Global Health, Perelman School of Medicine, University of Pennsylvania, Philadelphia, PA 19104, USA; 2 Department of Medicine, College of Medicine, Chang Gung University, Taoyuan 333, Taiwan; 3 Department of Pharmacology, College of Medicine, National Taiwan University, Taipei 100, Taiwan; 4 Department of Internal Medicine, National Taiwan University Hospital, Taipei 100, Taiwan; 5 Department of Dermatology, Chang Gung Memorial Hospital, Linkou, Taoyuan 333, Taiwan; 6 Drug Hypersensitivity Clinical and Research Center, Chang Gung Memorial Hospital, Linkou, Taoyuan 333, Taiwan; 7 Immune-Oncology Center of Excellence, Chang Gung Memorial Hospital, Linkou, Taoyuan 333, Taiwan; 8 Graduate Institute of Clinical Medical Sciences, College of Medicine, Chang Gung University, Taoyuan 333, Taiwan; 9 Cancer Vaccine and Immune Cell Therapy Core Laboratory, Chang Gung Memorial Hospital, Linkou, Taoyuan 333, Taiwan; 10 Department of Dermatology, Xiamen Chang Gung Hospital, Xiamen 361000, China; 11 School of Medicine, National Tsing Hua University, Hsinchu 300, Taiwan; 12 Department of Epidemiology, Harvard T.H. Chan School of Public Health, Boston, MA 02115, USA; 13 Department of Dermatology, Massachusetts General Hospital, Boston, MA 02114, USA; 14 Graduate Institute of Biomedical Electronics and Bioinformatics, College of Electrical Engineering and Computer Science, National Taiwan University, Taipei 106, Taiwan

**Keywords:** biomarker, cancer, cutaneous squamous cell carcinoma, diagnosis, extracellular vesicles, exosomes, microarray, malignancy, prognosis

## Abstract

**Simple Summary:**

Biomarkers including DNA, RNA, and surface-associated proteins in tumor-derived extracellular vesicles promote accurate clinical diagnosis and indicate the prognosis of cancer. In this systematic review, pre-clinical and clinical studies on extracellular vesicles derived from cutaneous squamous cell carcinoma (cSCC-derived EVs) were summarized, for which studies on the genomics, transcriptomics, and proteomics of cSCC-derived EVs were highlighted. The contents in cSCC-derived EVs may reflect the mutational landscape of the original cancer cells or be selectively enriched in extracellular vesicles, as provided by the significant role of target molecules including desmoglein 2 protein (Dsg2), Ct-SLCO1B3 mRNA, CYP24A1 circular RNA (circRNA), long intergenic non-coding RNA (linc-PICSAR) and DNA Copy Number Alteration (CNA). Evidence of these studies implied the diagnostic and therapeutic potential of cSCC-derived EVs for cutaneous squamous cell carcinoma.

**Abstract:**

Cutaneous squamous cell carcinoma (cSCC) as one of the most prevalent cancers worldwide is associated with significant morbidity and mortality. Full-body skin exam and biopsy is the gold standard for cSCC diagnosis, but it is not always feasible given constraints on time and costs. Furthermore, biopsy fails to reflect the dynamic changes in tumor genomes, which challenges long-term medical treatment in patients with advanced diseases. Extracellular vesicle (EV) is an emerging biological entity in oncology with versatile clinical applications from screening to treatment. In this systematic review, pre-clinical and clinical studies on cSCC-derived EVs were summarized. Seven studies on the genomics, transcriptomics, and proteomics of cSCC-derived EVs were identified. The contents in cSCC-derived EVs may reflect the mutational landscape of the original cancer cells or be selectively enriched in EVs. Desmoglein 2 protein (Dsg2) is an important molecule in the biogenesis of cSCC-derived EVs. Ct-SLCO1B3 mRNA, and CYP24A1 circular RNA (circRNA) are enriched in cSCC-derived EVs, suggesting potentials in cSCC screening and diagnosis. p38 inhibited cSCC-associated long intergenic non-coding RNA (linc-PICSAR) and Dsg2 involved in EV-mediated tumor invasion and drug resistance served as prognostic and therapeutic predictors. We also proposed future directions to devise EV-based cSCC treatment based on these molecules and preliminary studies in other cancers.

## 1. Introduction

Cutaneous squamous cell carcinoma (cSCC) is the second most common nonmelanoma skin cancer accounting for 20–50% of skin cancers with a rising incidence of about 5–13 times from 1976–1984 to 2000–2010 [1,2,3]. While typically remediable with prompt surgical removal, the sheer number of patients diagnosed with cSCC results in a substantial number of cSCC-related death [4,5]. The mutational landscape and risk factors beyond ultraviolet radiation of cSCC are not yet fully investigated, which may affect clinical decisions in precision medicine [6,7]. This is further complicated by the heterogeneity of tumor cells across time and subpopulations identified in traditional genetic analysis on tissue biopsies [8,9]. To elucidate the genetic and epigenetic complexity of cSCC, extracellular vesicles (EVs) have been incorporated in several approaches to providing clinicopathological parameters. EVs are small membrane-bound vesicles secreted by a variety of human cells, including tumor cells, that can be detected in blood, urine, saliva, and other bodily fluids [10]. The use of EVs has emerged as an attractive candidate for cancer liquid biopsy due to their availability, excellent stability, and resistance to disruption compared to circulating tumor cells (CTCs) and cell-free DNA (cfDNA) [10,11,12]. EVs contain selectively incorporated molecules from their parent cells, including proteins such as heat shock cognate 71 kDa protein (HSC70), CD9, and CD63), RNA (e.g., mRNA, miRNA, circRNA, and lncRNA), DNA, and lipids [10,13]. These luminal or surface molecules act through paracrine or autocrine signaling and are involved in cancer development, immunoediting, metastasis, and drug resistance [14,15,16]. In clinical and translational oncology, tumor-derived EVs and exosomes can be used to infer cancer diagnosis, prognosis, and recurrence [10,17,18,19,20,21]. Their potential as biomarkers for hepatocellular carcinoma (HCC), various pediatric solid tumors, pancreatic, ovarian, and bladder cancer has been shown in preliminary research [22,23,24,25]. Others have used EVs as prognostic indicators for colorectal cancer, breast cancer, and HCC [22,26,27]. More advanced applications of EVs include prediction and monitoring of treatment response as well as therapeutic cargos [28,29,30,31]. As so, EVs represent a promising tool in investigational dermatology [32]. In this review, current advances in the design, utility, applications, and clinical relevance of cSCC-derived EVs were summarized.

## 2. Materials and Methods

### 2.1. Literature Search

This study was done in accordance with the Preferred Reporting Items for Systematic Reviews and Meta-Analyses (PRISMA) [33], following the same protocol as described in previous studies [34,35,36,37]. A primary literature search was conducted with PubMed, Ovid/MEDLINE, and Cochrane Library databases on 14 May 2022, without limitation as to dates. Medical Subject Headings (MeSH^®^) controlled vocabulary, text words, and database-specific wildcards were utilized to develop the search terms. The full list of the search terms is available in online Supplementary Materials (Table S1).

### 2.2. Study Selection and Appraisal

All reviewers independently screened all article titles and abstracts to include clinical trials involving patients with cSCC or laboratory studies using human cSCC cell lines, written in English, or biomarkers in EVs for the diagnosis, prognosis, or treatment of cSCC. Studies on mucosal SCC, reviews, or articles not written in English were excluded. Rationales for exclusion and article appraisals were recorded at every stage. The final decision on study selection was reached by discussion. References of included and excluded studies were reviewed for potential studies not identified through the initial search strategy and added according to the criteria mentioned above.

### 2.3. Data Extraction and Analysis

Included studies were summarized using a data extraction form. Studies were graded using the Oxford Centre for Evidence-Based Medicine 2011 Levels of Evidence. Bias risk and methodological qualities were assessed using the National Institute Health Tool for clinical studies and the Quality Assessment Tool for In vitro Studies and preclinical studies.

## 3. Results

**Figure 1:**
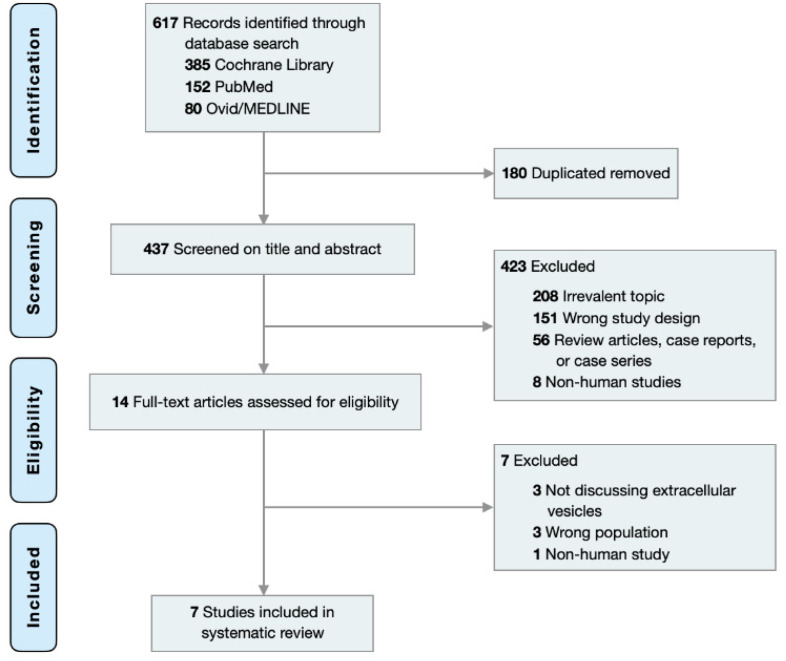
PRISMA flow chart for study selection.

Initially, 617 articles through 20 May 2022 were identified in Cochrane Central, PubMed, and MEDLINE with keywords and MeSH terms including (‘extracellular vesicle’ or ‘apoptotic body’ or ‘exosome’ or ‘microvesicle’) and ‘cutaneous squamous cell carcinoma’. 180 duplicates were removed and 423 of the remaining 437 articles were excluded after title/abstract screening. 14 articles were subjected to full-text review among which 7 were excluded. 7 studies were included in this systematic review (Table 1) as depicted by the PRISMA flow diagram (Figure 1). The PRISMA 2020 checklist and quality assessment of the included studies are available in online Supplementary Materials (Tables S2 and S3).

EVs were isolated from patient serum or culture media of human cSCC cell lines (3), including A431 (4), HSC-5 (1), and SCL-1 cells (1). All studies followed a standard sequential ultracentrifugation technique for EV isolation [44], and successful isolations were verified according to the International Society of Extracellular Vesicles guidelines using Transmission Electron Microscopy (TEM), Nanoparticle Tracking Analysis (NTA), and Western blotting for EV-associated proteins (e.g., CD9, CD63) [45]. One study used recessive dystrophic epidermolysis bullosa (RDEB)-associated SCC cells (RDEB-SCC cells) for disease-specific profiling. There were three studies that investigated EV-associated RNA [17,42,43], three studies on surface-associated proteins [17,38,40], and one study on EV-associated DNA [41].

**Figure 2:**
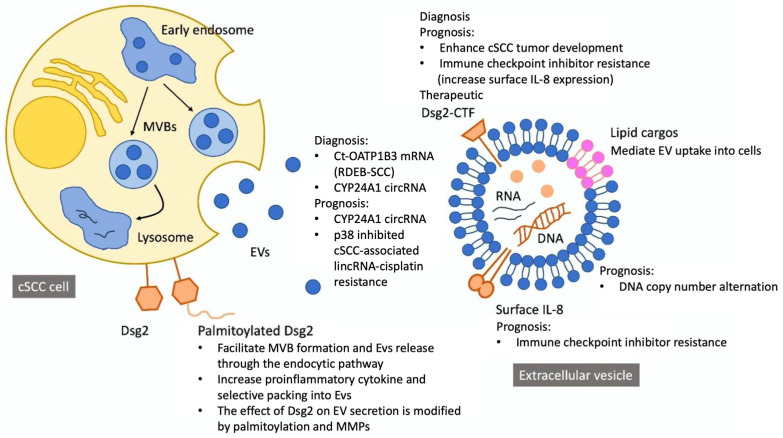
Contents and potential implications of cutaneous squamous cell carcinoma-derived extracellular vesicles.

EVs were further subdivided by their size and density. Four studies isolated small EVs (sEVs, or exosomes) [17,38,40,41], and the other three included both large EVs (lEVs, or microvesicles) and sEVs for further analysis [39,42,43]. Low-density (LD-EV) versus high-density (HD-EV) EVs were characterized in two studies [17,40]. LD-EVs (1.11–1.14 g/mL) and HD-EVs (1.26–1.29 g/mL) were separated by centrifugation in solutions with different iodixanol concentrations.39 These two EV categories demonstrate distinct physical and biological properties [46,47]. The aim of most studies was to develop EV-based liquid biopsy for the screening and diagnosis of cSCC [38,39,40,41], while EV-based prognostic biomarkers for therapeutic decision were also addressed in some studies [17,42,43] (Figure 2).

## 4. Discussion

### 4.1. Biogenesis of cSCC-Derived EVs

Desmoglein 2 (Dsg2) is a component of desmosomal cell–cell adhesion structure [48], and is present in all epithelial-derived cells with a variety of biological functions including epithelial-to-mesenchymal transition, cell proliferation, and migration [49,50,51]. Three articles suggested a critical role of Dsg2 in EV biogenesis, modulation, and biological functions [17,40,49].

Flemming et al. [17] found a two-fold increase in sEV release from A431-Dsg2/GFP tumor cells that overexpressed palmitoylated Dsg2. The effect of Dsg2 was mitigated by 50μM 2-bromopalmitate, an irreversible palmitoylacyltransferases inhibitor, and Dsg2cacs transfection, which abrogated Dsg2 palmitoylation. Furthermore, A431-Dsg2/GFP xenografts in immunocompromised mice resulted in significantly greater tumor growth and higher plasma sEV levels, and a single dose of 20 μg Dsg2 modulated sEVs was able to enhance the tumorigenic potential of A431-GFP xenografts. Overmiller et al. [38] confirmed that Dsg2 overexpression increased sEVs secreted per cell and EV-associated protein density. Besides, the effect was blunted by transducing A431-Dsg2/GFP cells with shRNA targeting Dsg2. Apart from fatty acid modifications, the role of post-transcription Dsg2 proteolysis was further assessed. They discovered a unique Western blot signal at ~65 kDa in EVs isolated from primary human keratinocytes, which corresponded to the intracellular C-terminal fragment (CTF) of Dsg2 and hypothesized that full-length Dsg2 was modified by matrix metalloproteinase (MMP), such as metalloproteinase 17 (ADAM17), into a ~95 kDa secreted ectodomain and an intracellular CTF domain that was responsible for EV modulation. Indeed, EV biogenesis was blunted by the addition of broad-spectrum MMP inhibitors. Therefore, post-translational Dsg2 modification would be a powerful leverage point for EV-based characterization and treatment of cSCC.

### 4.2. Diagnostic Value of cSCC-Derived EVs

EV-based liquid biopsy had been an active field of oncology research in the past decade. A study involving 139 patients with early stage pancreatic, ovarian, or bladder cancer demonstrated an area under the curve of 0.95 (sensitivity 71.2%, specificity 99.5%) using an extracellular vesicle protein-based diagnostic blood test [25]. A meta-analysis of 39 studies on pancreatic cancer concluded that EV RNAs were effective biomarkers for pancreatic cancer screening with 79% sensitivity and 87% specificity, even at its rudimental stage [52]. When combining both protein and RNA biomarkers, they came to an excellent diagnostic performance of 90% sensitivity and 94% specificity for stage I and II pancreatic cancer. EV RNA also allowed detection of non-small cell lung cancer and differentiation from small cell lung cancer [53]. We identify two potential RNA molecules (Ct-SLCO1B3 and circ-CYP24A1) with such potential:

#### 4.2.1. Ct-SLCO1B3 (Ct-OATP1B3 mRNA)

Patients with RDEB are prone to aggressive cSCC due to repetitive injuries to the skin. Therefore, it is important to screen for cSCC in this population. Sun et al. [39] analyzed the expression of tumor marker gene Ct-SLCO1B3 in EVs from cultured RDEB and non-RDEB tumors. Ct-SLCO1B3 was exclusively expressed in RDEB-SCC-derived EVs but not in those derived from non-cancerous RDEB keratinocytes. An in vivo study showed that Ct-SLCO1B3 transcriptions were only present in EVs isolated from RDEB tumor-bearing mice who received RDEB-SCC2 cell xenografts [39]. Collectively, these findings suggested Ct-SLCO1B3 in EVs as a promising diagnostic biomarker for cSCC in patients with RDEB. More investigations on the generalizability of Ct-SLCO1B3 in EVs for non-RDEB patients with cSCC are warranted.

#### 4.2.2. Circ-CYP24A1

Zhang et al. [42] employed RNA-sequencing (RNA-seq) for exosomal circular RNA (circRNA) profiling collected from the serum of five patients with cSCC. A total of 7577 circRNAs were detected, of which 25 were up-regulated and 76 were down-regulated in cSCC, compared to healthy subjects by more than two-fold. These circRNAs constituted two unique RNA clusters: genes mediating T cell and NK cell cytotoxicity and MHC protein complex were enhanced whilst genes regulating central carbon metabolism, cellular component organization, and cell cycle decreased. Among the first cluster, circ-CYP24A1 was increased to the greatest extent. Because the circular structure confers resistance to RNase, circ-CYP24A1 was suggested to be an ideal biomarker for cSCC [42].

Although cSCC are usually identified by full-body skin examination and confirmed by biopsy, such practice may not always be feasible or may be time-consuming. Incorporating EV-based liquid biopsy for screening and differentiation in certain high-risk patients may facilitate early detection and avoid unnecessary biopsy.

### 4.3. Prognostic Value of cSCC-Derived EVs

The most important aspect of modern medicine is precision medicine. EV characteristic profiles had been postulated to provide insights into tumor behaviors. Previous research showed that PD1 and PD-L1 expression on melanoma-derived EVs predicted resistance to checkpoint inhibitors [54]; EV mRNA could predict NSCLC survival and efficacy of different treatment modalities [53]; long noncoding RNA may serve as a prognostic and immunotherapeutic predictor for testicular germ cell tumor and HCC [55,56]. Besides, EV DNA reflected both chromosomal and mitochondrial DNA of the original tumor, which could be a powerful tool to assess tumor genome and predict prognosis [57]. Here, we presented one biomarker of each class (protein, RNA, DNA) that possessed prognostic values for cSCC.

Flemming et al. [17] demonstrated a large reduction in miR146a-5p (miR146a) in A431-Dsg2/GFP cells and associated sEVs compared to A431-GFP cells. Since miR146a inhibited the NF-ĸB proinflammatory pathway, lower miR146s led to increased IL-8 expression on EV surfaces [58]. To assess the effect of IL-8 on immune checkpoint inhibitor [59,60,61] resistance, the researchers measured plasma IL-8 levels before and after nivolumab treatment in patients with head and neck SCC, and they found that high circulating IL-8 levels were associated with low responsiveness to immunotherapy. In their follow-up study, Flemming et al. [40] found that increased Dsg2 expression on cSCC-derived EVs was associated with lower EV density. Moreover, chemoattractants such as IL-8, GRO⍺, and IL-1⍺ were enriched on these LD-EVs. Therefore, EV Dsg2 expression and its downstream surrogates including LD-EV/HD-EV ratio and surface IL-8 density could be prognostic biomarkers for anti-neoplastic treatments including immunotherapy.

### 4.4. p38 Inhibited cSCC-Associated Long Intergenic Non-Coding RNA (linc-PICSAR)

Wang et al. [43] explored the role of lnc-PICSAR in patients with cisplatin-resistant cSCC and HSC-5 cells. A significantly increased amount of lnc-PICSAR in EVs derived from cisplatin-resistant cSCC patients and cell lines was observed, as compared to healthy individuals and cisplatin-susceptible HSC-5 cells. lnc-PICSAR was directly involved in cisplatin resistance through miR-485-5p inhibition, which in turn promoted REV3L gene expression. Therefore, the concentration of lnc-PICSAR in serum-derived EVs from patients with cSCC could guide the selection of chemotherapy regimens [42].

#### 4.4.1. Circ-CYP24A1

Apart from its diagnostic value, circ-CYP24A1 showed a marginally positive correlation with tumor thickness (Pearson r = 0.8689, *p* = 0.0558) [42]. The authors hypothesized a tumerogenic role of aberrantly expressed EV circ-CYP24A1 in cSCC, as supported by an observed uptake of circ-CYP24A1 by A431 and SCL-1 cells co-cultured with PKH67-labeled EVs.34 Furthermore, EVs transfected with si-circ-CYP24A1, which knockdowned circ-CYP24A1, restrained proliferation, migration, and invasion of cSCC cells and induced their apoptosis. Given these finding, circ-CYP24A1 was suggested to be a marker for cSCC prognosis [42].

#### 4.4.2. DNA Copy Number Alteration (CNA)

Nguyen et al. [41] performed low-coverage whole-genome sequencing of fixed paraffin-embedded (FFPE) and serum EV DNAs from two patients with metastatic cSCC and three patients with metastatic tongue base SCC. Compared to mucosal SCC, cSCC showed more CNAs (25 versus 11.3 regions; 1.225 × 10^9^ versus 4.433 × 10^8^ basepairs) and more overlaps with FFPE CNAs (16 versus 3 regions; 3.25 × 10^8^ versus 3.267 × 10^8^ basepairs). Although the majority of CNAs were deletions, areas of duplication in EV-DNA may be more reflective of mutations in FFPE-DNA. Duplications in Chr 7p, 8q, and 20q were found in both samples and were consistent with the published mutational landscape of aggressive cSCC [62]. However, because EV-DNA was derived from both tumoral and non-tumoral DNA, there was considerable heterogeneity in FFPE and EV-DNA correlation among different patients. Hence, whether CNA profiles of EVs may be used to accurately detect metastatic SCC should be validated through more studies.

Although surgical excision is the definite treatment, systemic chemotherapy or immunotherapy is usually needed for advanced cSCC and metastatic disease. Even in localized cSCC, subgroups with metastatic spreading ability are not uncommon, and adjuvant systemic treatment may sometimes be necessary [63]. Furthermore, re-biopsy to assess emerging drug resistance is difficult. Given its dynamic changes and availability, the application of EV-based technology in precision cSCC management is endless.

### 4.5. Therapeutic Potential of csCC-Derived EVs

Covalent and non-covalent modification of EV membrane molecules could enhance specific binding to target cells [64]. These externally modified EVs had been used to deliver therapeutic drugs in glioblastoma, breast cancer, and colorectal cancer models [65,66,67]. In the same study by Overmiller et al. [38], when human fibroblasts were co-cultured with A431-Dsg2/GFP cells, 55% more fibroblasts became GFP positive than with A431-GFP cells, suggesting that the uptake of sEV may be facilitated by Dsg2. Thus, EV Dsg2 modification may improve treatment delivery to cSCC microenvironment. The ability to target cancer cells and transference of genetic materials had been studied to reverse oncogenic mutations. miRNA in human macrophage-derived EVs had shown anti-HCC [68,69] and anti-glioma activity [70]. Given the importance of Dsg2 in cSCC-derived EV biogenesis and functioning [17,38,40] miRNA targeting Dsg2 translation is promising.

## 5. Conclusions

Biomarkers including DNA, RNA, and surface-associated proteins in tumor-derived EVs promote accurate clinical diagnosis and indicate prognosis of cancer. The diagnostic and therapeutic potential of cSCC-derived EVs have been addressed in studies focusing on target molecules including Ct-OATP1B3 mRNA, circ-CYP24A1, linc-PICSAR, and DNA CNA. Due to limited experimental data, further studies on the role of EV in the pathophysiology of cSCC and clinical relevance of cSCC-derived EVs are required to establish the clinical utility of EVs in the diagnosis and management of cSCC.

## Figures and Tables

**Table 1:** Characteristics of included studies.

Study	Target Molecule	Source and Size of EV	Key Findings
**EV-Based Diagnostic Biomarkers for cSCC**			
Overmiller 2017 [38]	Dsg2-CTF	- A431 cells with and without Dsg2/GFP transfection (in vitro) - sEV	Dsg2 was cleaved by MMP and the intracellular Dsg2-CTF was bound to EVs. Soluble Dsg2 ectodomain and full-length Dsg2 were released into TCL. Dsg2-CTF increased EV biogenesis through Cav1 endocytosis. Dsg2-CTF facilitated EV transfer from keratinocytes to fibroblasts. Dsg2-CTF increased P-EGFR and downstream P-AKT in fibroblasts cultured with SCC EVs, leading to fibroblast proliferation.
Sun 2017 [39]	Ct-SLCO1B3 (Ct-OATP1B3 mRNA)	- RDEB-SCC cells (in vitro and in vivo) - lEV & sEV	Ct-SLCO1B3 transcripts were only found in RDEB-SCC-derived EVs but not in those from non-tumor RDEB or normal human keratinocytes. Ct-SLCO1B3 transcripts were only found in serum EVs from RDEB tumor-bearing mice but not in those isolated from control serum.
Flemming 2021 [40]	- Surface-associated cytokines - sEV density	- A431 cells with and without Dsg2/GFP or Dsg2cacs/GFP transfection (in vitro) - sEV	Dsg2cacs increased the percentage of HD-sEVs subpopulation. High levels of surface RANTES, GRO⍺, PDGF-AB/BB, VEGF, G-CSF, IL-8, MDC, IL-1⍺, and FGF-2 on A431-derived sEVs. Dsg2 increased IL-8, IL-1⍺, RANTES, GRO⍺ on sEVs. Dsg2cacs increased IL-6, IL-15, G-CSF, GM-CSF, PDGF-AB/BB on sEVs. cSCC-derived sEVs contributed to their immune evasion, prooncogenic, and proangiogenic activity.
Nguyen 2021 [41]	DNA CNA	- Serum of 2 patients with metastatic cSCC - sEV	Overall CNA correlation between FFPE and EV-associated DNA was weak, and CNA was under-represented in the EV-DNA based analyses. Concordant regions of duplication were found in Chr 7q, 8q, and 20q.
**EV-based prognostic biomarkers for cSCC**			
Zhang 2021 [42]	circRNA	- Serum of 5 cSCC patients; A431 and SCL-1 cells (in vitro) - lEV & sEV	25 circRNAs were up-regulated and 76 were down-regulated by more than two-folds in patient EVs compared to healthy subjects. Up-regulated circRNAs were mainly involved in T cell and NK cell-mediated cytotoxicity and MHC protein complex. Down-regulated circRNAs were mainly related to central carbon metabolism in cancer, cellular component organization, and cell cycle. circ-CYP24A1 was highly elevated in patient EVs while circ-ALDH3A2 was decreased. circ-CYP24A1 had marginal correlation with tumor thickness (Pearson r = 0.8689, *p* = 0.0558); circ-ALDH3A2 was significantly correlated to pre- and post-op serum SCC-Ag (r = −0.9025 and -0.9388, *p* = 0.0360 and 0.0180). EVs transferred circ-CYP24A1 into cSCC cell lines, which promoted tumor proliferation, migration, invasion, and inhibits apoptosis.
Flemming 2020 [17]	- Surface-associated cytokines - miRNAs	- A431 cells with and without Dsg2/GFP or Dsg2cacs/GFP transfection (in vitro and in vivo) - sEV	Dsg2 increased endocytosis and decreased MVBs degradation by lysosomes, which increased sEV biogenesis. Dsg2 increased I-TAC, IL-8, MIP-1B, ANGPT2, CCL28, BTC, EGFR, TRAIL (R4), IGFBP-3, Dtk, and ICAM in sEVs while TIMP1 and 2 decrease. Dsg2 downregulated miR 146a, which inhibited the NF𝒦B proinflammatory pathway including IL-8 synthesis. Overexpression of palmitoylated Dsg2 on A431-derived sEVs enhanced SCC tumor growth and a single dose of SCC-derived sEVs overexpressing Dsg2 was capable of inducing tumorigenesis. Patients with higher baseline IL-8 levels were less responsive to checkpoint inhibitors (Nivolumab).
EV in the treatment of cSCC			
Wang 2021 [43]	Lnc-PICSAR	- Serum of 30 cSCC patients; cisplatin-resistant HSC-5 cells (in vitro and in vivo) - lEV & sEV	lnc-PICSAR was elevated in EVs derived from cSCC patients’ serum and HSC-5 cells. lnc-PICSAR was further elevated in cisplatin-resistant HSC-5 cells.

sEVs = small extracellular vesicles; lEV = large extracellular vesicles; HD = high density; Dsg2 = desmoglein 2; GFP = green fluorescence protein; cSCC = cutaneous squamous cell carcinoma; RDEB = recessive dystrophic epidermolysis bullosa; MVBs = multi-vesicular bodies; miRNAs = microRNA; TCL = total cell lysate; CTF = C-terminal fragment; MMP = matrix metalloproteinase 17; P-EGFR = phosphorylated EGFR; P-AKT = phosphorylated AKT; CAN = copy number alteration; FFPE = formalin-fixed paraffin-embedded tissue sample; lnc-PICSAR = long noncoding RNA p38 inhibited cutaneous squamous cell carcinoma-associated lincRNA; circRNA = circular RNA; op = operation. Furthermore, applicable to treatment selection.

## Data Availability

The dataset supporting the conclusions of this article is included within the article. Search strategies are available on searchRxiv.

## Supplementary Materials

The following supporting information can be downloaded at: https://www.mdpi.com/article/10.3390/cancers14205098/s1. Table S1: Keywords and MeSH terms used in different databases in the literature search; Table S2: PRISMA 2020 checklist; Table S3: Quality assessment of included studies.

## Abbreviations

Cutaneous squamous cell carcinoma (cSCC), circulating tumor cells (CTCs), cell-free DNA (cfDNA), extracellular vesicles (EVs), short hairpin RNA (shRNA), intracellular C-terminal fragment (CTF), matrix metalloproteinase (MMP), circular RNA (circRNA), long intergenic non-coding RNA (lincRNA), fixed paraffin-embedded (FFPE), DNA copy number alteration (CNA), circular RNA (circRNA).
